# Implementation Process of General Land-Use Planning and Its Adjustment—A Case Study of Rongchang District in Chongqing, China

**DOI:** 10.3390/ijerph18115639

**Published:** 2021-05-25

**Authors:** Lingli Li, Jinjie Wang, Chaoxian Yang, Chaofu Wei

**Affiliations:** College of Resources and Environment, Southwest University, Chongqing 400715, China; lilingli2020@foxmail.com (L.L.); wjjhty2021@163.com (J.W.); yangcx@swu.edu.cn (C.Y.)

**Keywords:** Rongchang District, general land-use planning, implementation process, adjustment

## Abstract

General land-use planning has a critical role and a guiding significance for economic and regional social development. To increase the ability of planning to cope with regional economic changes in an orderly manner and to defend the legal status of the planning guidance role, this article takes Rongchang District as a case study. This study combines GIS spatial analysis to select speed indicators, the center of gravity offset theory, and the spatial fit model and analyses the implementation process and the adjustment situation of planning from the perspectives of ‘quantity’, ‘time’, and ‘space’. The main results are as follows: (1) The completion rate of cultivated land and the surplus rate of urban land show that planning can effectively guide the direction of land use, with the results of 101.9% and 15%, respectively. The difficulty of planning implementation lies in the control of rural residential land because the actual annual average withdrawal rate of rural residential land is less than one-third of the planned rate, with an actual withdrawal area of 97.22 hm^2^ per year on average. (2) The results of the spatial coincidence degree and the barycentric offset angle demonstrate that planning plays a prominent role in guiding the direction of land use, although deviations remain between planned and actual land-use demand, with values ranging from 0.9 to 1 and an angle of less than 30° between the implementation process and the target year. (3) From 2013 to 2015, the planning of the study area was adjusted 32 times with an area of 2301.7 hm^2^. This finding indicates that planning is characterized by frequent disorder and that the gap between land use and planning is alleviated at the cost of the planning authority. (4) The degree of the coincidence between the adjusted plan and the land-use change data decreased year by year, reaching 0.99 two years after implementation of the plan, which is closest to the actual land demand. Thus, general land-use planning can guide the direction of land use to some extent, and the adjustment of planning can alleviate the contradictions of land-use demand under the changes of economic development, but the disorder ignores the legal status of planning. Making regular dynamic adjustments to the plan can provide ideas for planning compilation and revision while maintaining economic benefits and guiding functions without losing legal status.

## 1. Introduction

General land-use planning is a general arrangement for the development, utilization, consolidation, and protection of land in a certain period and region. It is an important part of land management [1] and includes characteristics of economy and law. Planning, a basic premise of land-use control in China [2], is beneficial to the coordinated development of economy, society, and environment and plays a crucial role in guiding the direction of land use in China [3]. The Law of the People’s Republic of China on Land Administration stipulates that the implementation of general land-use planning should restrict land transformation from agricultural use into construction use and strictly protect cultivated land, thus establishing its legal status. In practice, some places adjust their planning scheme almost every year [4], considering the premise of economic development only [5], which seriously affects the planning authority [6,7] and fundamentally affects progress in the rule of law in land management. The legal status of planning is in jeopardy.

Rational processes are inapplicable when addressing complex societal issues [8], so the timely adjustment of the plan is the performance of flexibility and dynamics, which is conducive to strengthening the ability of planning implementation. Currently, there is a consensus in planning circles on the recognition of the dynamic nature of planning [6,9,10,11,12,13], which can develop the vitality of planning and narrow the gap between planning and the land-use status quo. On the one hand, scholars have discussed the uncertainty of planning during the preparation, approval, implementation, and planning monitoring stages [14,15]. At the same time, strategic ideas [11,16,17,18] have been integrated into the study of land-use planning [19,20,21], such as increasing planning flexibility [22] and establishing a target framework. On the other hand, scholars have explored improvements to land-use planning methods. These improvements include implementing rolling planning by predicting short-term behavior [23], increasing flexibility while maintaining the rigidity of general land-use planning [24], implementing double-layer planning combining certainty and uncertainty, and developing a multi-objective programming model [25]. Finally, with regard to the implementation of dynamic planning, scholars propose that the ‘dynamic’ is actually a constrained ‘dynamic’ [26]; it is necessary to regularly adjust and evaluate the implementation of the planning [27] and take the evaluation results as a dynamic adjustment basis for planning compilation [28,29]. Some planning implementation discussions consider the legality, strategy, and ‘uncertainty’ in foreign countries [30,31,32,33]. These studies mainly focus on evaluations of planning objectives; implementation effects based on the degree of consistency [4,34,35]; and impact assessments before, during and after implementation [36,37]. In recent years, researchers in China have actively explored evaluation theory [26], index systems, and evaluation methods [38], which has laid the foundation for the monitoring, evaluation, and management framework system of planning implementation.

Therefore, existing research on the gap between land-use planning and reality usually focuses on evaluating the implementation effect of planning and neglects the mutual influence between the guiding role of planning and its adjustment. This paper evaluates the implementation process of general land-use planning and its adjustment to identify the connection between planning adjustment and the effect of planning implementation. Furthermore, this study chooses a reasonable time interval to narrow the gap between planning and reality in an orderly and standard way. Consequently, without violating the authority and seriousness of planning, this study can control the actual situation and better guide social and economic development.

This paper has two main purposes: (1) to recognize the contribution of planning adjustment to the guiding role of land-use planning and (2) to fix the time point of dynamic planning adjustment and reduce the frequency of general land-use planning adjustment.

## 2. Materials and Methods

### 2.1. Study Area

**Figure 1:**
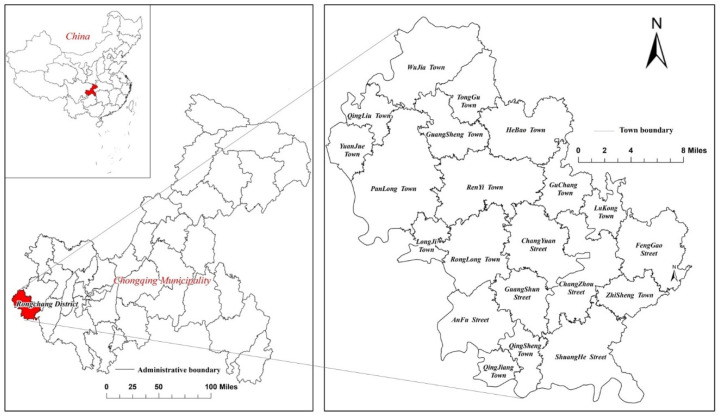
Location of Rongchang District.

Rongchang District (29°15′~29°41′ N, 105°17′~105°44′ E) is located in the western Chongqing Municipality, China (Figure 1). It is the strategic heart of the CCEZ (Chengdu-Chongqing Economic Zone), the fourth largest economic zone in China. The study area is dominated by shallow hill topography, and the local geomorphic types are mainly characterized by terraces, floodplains, and box low mountains. It is a typical hilly area, with the average altitude of the area ranging from 300 to 400 m [39]. The terrain of Rongchang is high in the south and low in the north and gradually slopes from the northeast to the southwest, where most of the localities are flat to slightly undulating [40]. The region has a typical subtropical monsoon humid climate, with a temperate climate, abundant rainfall, high humidity, little frost and snow, and low wind. The annual average temperature in the area is approximately 17.80 °C, the annual total accumulated temperature is approximately 6482 °C, the average annual frost-free period is more than 327 days [41], the average annual sunshine duration is 1282 h, and the average annual rainfall is more than 1111.8 mm [39], which is mainly concentrated in summer. There are 151 rivers of different sizes in the district, most of which belong to the Tuojiang River system, with a total water run-off of 325 million m³ [42]. By the end of 2015, there were 701,000 permanent residents, with an urbanization rate of 51.16% [43]. It comprises an area of 1076.78 km^2^, including six streets and 15 towns, 209 administrative villages, and 42 community units. This area is a national modern agricultural demonstration zone determined by the State Council [41]. Agricultural land constitutes the greatest proportion, representing 83.12% of all land-use types in the region, whereas construction land accounted for 15.7% and other land accounted for 1.18% as of the end of 2015 (Table 1). 

### 2.2. Data Collection and Method Analysis

#### 2.2.1. Data Source

Land-use change investigation data from 2009 to 2015 were adopted for the present land-use data. The adjustment data on the general land-use planning came from the revised project statistics table (Appendix A, Table A1), and the land-use planning data came from The General Land-Use Planning of Township in Rongchang District, Chongqing Municipality (2009–2020) and its special reports, the work report of “Four Checks and Four Comparisons” in Rongchang District (2011–2015) and The General Land-Use Planning of Rongchang District, Chongqing Municipality (2006–2020). The social and economic data came from the Statistical Yearbook of Rongchang District.

The General Land-Use Planning of Rongchang District, Chongqing Municipality (2006–2020) was compiled in 2010, and the planning period was from 2006 to 2020. The planning implementation period selected in this paper is from 2009 to 2015. The change in land-use structure in Rongchang District during the evaluation period and the planning control target in 2020 are shown in Table 1.

#### 2.2.2. Indicators Method

This paper selects cultivated land, urban industrial and mining land, and rural residential land to conduct a comprehensive analysis of the implementation process of planning (2009–2015) from the perspectives of quantity and time. Speed indicators, including the expansion matching degree and index use progress (Table 2), were selected to analyze the implementation of land planning for different land types.

#### 2.2.3. The Gravity Centre Index Method

Theoretically, the evaluation of planning implementation focuses on changes in quantity and spatial location. Therefore, this paper, which is based on GIS technology, has wider applicability due to the use of methods for the degree of spatial coincidence and the gravity center shift from the perspective of space.

There are three main gravity center indexes, namely, the regional center of gravity, the moving distance of the center of gravity, and the moving direction of the center of gravity [45]. The regional center of gravity indicates the “weight” of geographical objects in the spatial layout. The moving track can be obtained by connecting the adjacent time periods [46], which represents the direction of the key construction of the spatial layout. The degree of spatial compliance of the planning implementation can be judged by comparing the direction of the general land-use planning with the moving track direction [47]. Equation (1) is as follows (Equation (1)):

(1) $$x=\frac{\sum_{i=1}^{n}N_{i}P_{i}}{\sum_{i=1}^{n}N_{i}}\;y=\frac{\sum_{i=1}^{n}N_{i}Q_{i}}{\sum_{i=1}^{n}N_{i}}$$

where (x, y) are the coordinates of the center of gravity of the area in the spatial layout [48], Ni is the area of the research object, and Pi and Qi are the center coordinates of the I-th land-use unit.

Therefore, this paper uses the theory of gravity center shift to comprehensively analyze the urban industrial and mining land and rural residential land from the perspective of spatial layout [15]. Based on the spatial information management function of GIS, the database of construction land information management in the research area of the basic year, the evaluation year, and the planning year is established. By overlaying a 20 m × 20 m grid with the construction land data of each year, a grid with an area greater than 100 m^2^ is selected as the evaluation unit, and the central coordinates of each unit are obtained by using GIS spatial analysis tools. The coordinate value is input into Equation (1) to obtain the center of gravity coordinates of construction land in the required years. The consistency between the development trend of the evaluation year and the planning development trend can be judged according to the distance and deviation angle of the center of gravity movement [49].

#### 2.2.4. Metric of Spatial Layout Fit 

The spatial fit measurement model can judge whether the development trend of phased achievements is consistent with that of the planning goal in space. It can measure the degree of coincidence between the planning implementation process and the phased results after the implementation of the planning and planning objectives. The method is shown in Table 3.

(1) Degree of coincidence of the planning implementation process

Assuming that the land-use types in the entire study area are X and Y, the land-use change data from the base year to the end of the evaluation year are obtained by using the GIS spatial analysis function tool. “X” → “X” or “Y” → “Y” indicates that the land types remain unchanged, and “X” → “Y“ or “Y” → “X” indicates the land types have changed. By judging the area that is not in accordance with the planning goals in the changed plots, the spatial coincidence degree of each land-use spot can be obtained. 

Therefore, the land-use data in the basic year (2009) and the evaluation year (2015) are superimposed to screen out the land areas that did not meet planning purposes in different land types. Then, the value is input into Equation (2) in Table 3 to acquire the coincidence degree of each land type in the process of planning implementation.

(2) Degree of coincidence of phased results of planning

Assuming that A is a certain land-use type, there are three situations of area changes for land-use type A in a certain period: ① increase, ② unchanged, or ③ decrease. Thus, the area changes of land type A from the planning base year to the planning evaluation year (T1–T2) and from the planning evaluation year to the planning target year (T2–T3) can be expressed by ①, ②, and ③, respectively. By superimposing the present land-use status in the evaluation year (2015) with general land-use planning, the implementation and spatial distribution differences of each land-use type at the time of the assessment can be obtained.

There are a total of 9 types of situations after superimposing by GIS spatial analysis technology: “①–①”, “②–②”,and “③–③” indicate that the current situation of land use in the assessment year is consistent with the planning objectives, “②–①”and “②–③” indicate that the land use has not been changed in accordance with planning objectives in the assessment year, and “①–②”, “①–③”, “③–①”, and “③–②” indicate that the current situation of land use in the assessment year is not consistent with the planning objectives.

Therefore, the degree of spatial consistency of different types can be obtained by overlaying the data of land-use status in 2015 and the data of general land-use planning of 2020 after the adjustment of the planning in Rongchang District and counting the area of the plots that are not implemented in the planning and are not in line with the planning purpose.

Based on the land-use change survey data from 2012 to 2015, the land-use change data of each year are obtained through the function of GIS spatial superposition, where the urban construction land type is selected. The consistency between the urban land change data over the years and the urban land adjustment data from 2013 to 2015 are calculated to obtain the results.

## 3. Results and Analyses

### 3.1. Evaluation of the Implementation Process of General Land-Use Planning

#### 3.1.1. The Perspective of Quantity 

In the context of industrialization and rapid urbanization, a series of inharmonious phenomena have emerged, e.g., excessive emphasis placed on the speed and scale of urban construction, frequent breakthroughs in planning objectives at the scale of urban expansion, and massive occupation of cultivated land and ecological space [37,51,52]. In 2015, the total area of cultivated land was 59,381.77 hm^2^, which was 749.93 hm^2^ and 11,100.08 hm^2^ more than the area in 2009 and the controlled area in the planning year (2020), respectively. The target completion rate reached 101.89% (Table 4), which means that the protection strategy of cultivated land from the land management level was effectively implemented and the basic status of agriculture was guaranteed.

With the acceleration of industrialization and urbanization in China, large numbers of migrant workers leave their homes and become long-term urban residents. Therefore, it is an inevitable development trend that the size of cities and towns increases and the rural residential area decreases. In 2015, the area of town land reached 3046.46 hm^2^ in Rongchang District, which was 803.57 hm^2^ more than that in 2009. Compared with the planning control scale, there was an unused land area of 537.23 hm^2^, and the index surplus rate was approximately 15%, indicating that there was still a certain development space for urban land in the next five years and that, to a certain extent, the scale of urban land expansion was effectively controlled by planning.

In 2009, the area of rural residential land in the study area was 11,711.83 hm^2^, and planning estimated that rural residential land would be reduced to 8616.56 hm^2^ (Table 4). Under ideal circumstances, the annual withdrawal rate would be 281.39 hm^2^ per year. However, by the end of 2015, the area of rural residential land in Rongchang District was 11,128.5 hm^2^, with an average annual withdrawal rate of 97.22 hm^2^ per year, and there were still 2511.94 hm^2^ to be withdrawn. At the current exit rate, rural residential areas will surpass planning control in the planning target year, indicating that rural residential land will become a difficulty in achieving the planning target. The area of urban and rural construction land reached 14,459.35 hm^2^ in the entire region in 2015, which surpassed planning control (Table 1). The expansion of urban land has been controlled to a certain extent, while the exit rate of rural residential land is less than one-third of the ideal exit rate. Thus, the low withdrawal efficiency of rural residential land caused the excess of the planning goal for urban and rural construction land.

Therefore, in the following planning implementation process, it is essential to speed up the withdrawal of rural settlements, strengthen the control of the intensity of construction land expansion intensity, strictly control the new town land indicators, and improve the level of intensive use of land resources. This will achieve the goal of optimizing the structure of land use, promoting industry prosperity, increasing farmers’ income, meeting rural development needs, and promoting rural revitalization.

#### 3.1.2. The Perspective of Time 

The values were 1.26%, −5.24%, and 26.38% for the expansion intensity of cultivated land, rural residential land, and urban land, respectively, during the evaluation period based on an analysis of the expansion intensity index (Table 5). The gap in land expansion intensity between rural residential areas and urban areas was five times as high, indicating that urban construction in the study area was developing rapidly during the evaluation period, which was consistent with the overall economic development trend of the country and local areas. The index use progress of agricultural residential land withdrawal was only 18.85% and that of urban industrial and mining land was 59.93%, which is approximately three times that of rural settlements. Therefore, a contradiction exists between the large number of idle rural homesteads and urban construction land with gradually reduced development potential. During the assessment period of Rongchang District, an agricultural population of approximately 76,500 moved to cities, which is in contrast to the relatively small amounts of rural settlements withdrawn. This indicates a phenomenon of population flow without changes to the place of residence, which is also one of the reasons why the control of rural settlements in planning is difficult to implement. Therefore, attention should be given to the intensive effect of urban land use for promoting the sustainable development of the local economy without surpassing the indicators in the future. At the same time, the development of ideological work on the concept of non-agricultural population homestead withdrawal should be strengthened.

#### 3.1.3. The Perspective of Spatial Layout 

(1) Trajectory analysis of space gravity center shift

The center of gravity coordinates of construction land in the required years of 2009 (35,548,948.47, 3,261,256.17), 2015 (35,549,369.19, 3,260,841.34) and 2020 (35,549,730.14, 3,259,712.14) was obtained by using the gravity center index method.

**Figure 2:**
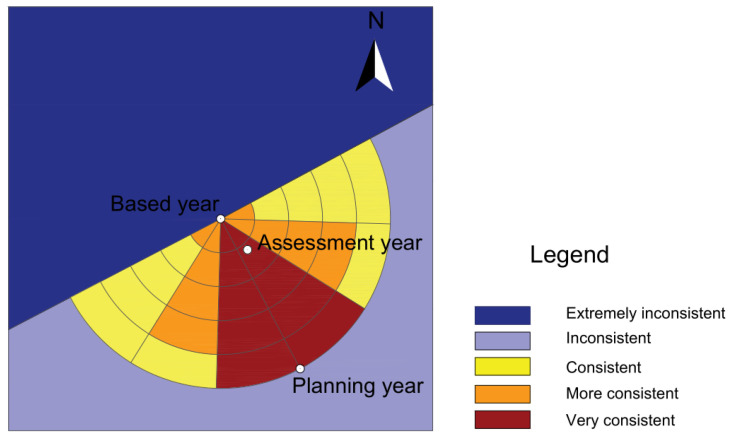
Spatial gravity center transfer of construction land in Rongchang District in 2015.

Thirty-two vector components can be obtained by setting the construction land central gravity coordinates in 2009 as the center of a circle and the offset distance from 2009 to 2020 as the radius, which is divided into five equal parts, and dividing the sector at an angle of 30°. At any point in those areas, the longer the distance of the center of gravity movement, the longer the implementation time of the planning. The smaller the deviation angle between the trajectories of the center of gravity movement and the planning offset, the more consistent is the direction of the planning (Figure 2).

The shift direction of the center of gravity of construction land in Rongchang District is 28° south by east in the planning year (2020), and the distance of the central gravity movement in 2015 stays within the expected distance in the planning year, which is located in the vector component of “very consistent”. Therefore, we can conclude that the development trend of construction land in 2015 is consistent with the planning development trend. The evaluation result of the spatial gravity shift of construction land in Rongchang District in 2015 was “very consistent”, indicating that the control measures of the spatial use of construction land have been effectively implemented, in line with general land-use planning at the macro level.

(2) Analysis of the spatial consistency of the planning implementation process

**Figure 3:**
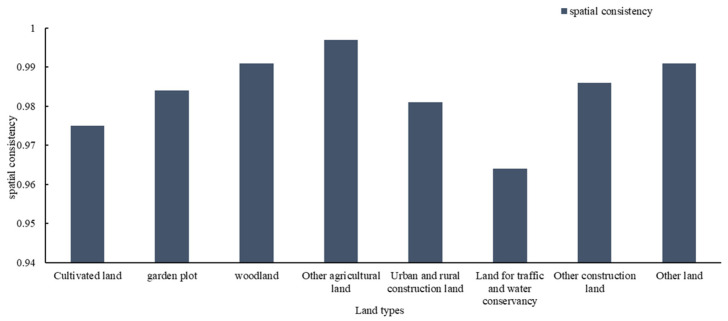
The spatial consistency of different types in the implementation process of the Rongchang District plan in 2015.

The coincidence degree of each land type in the process of planning implementation are shown in Figure 3.

The new Land Administration Law in China clearly stipulates that China implements a land-use regulation system and that the acts of utilizing land in disagreement with requirements should be punished after the use of land is specified in general land-use planning. A coincidence degree of less than 1 of each land type indicates that the implementation situation does not conform to the planning goal (Figure 3). On the one hand, the deviations during the evaluation period show that there are still illegal acts of transformation of land use without prior approval. On the other hand, those acts indicate that the law enforcement supervision of relevant departments is not sufficient.

In summary, the finding that the angle was less than 30° in 2015 between the center of gravity of construction land and the planned trajectory in Rongchang District and that the spatial coincidence was in the range of 0.9–1 for the planning implementation process demonstrates that the guiding role was prominent and that planning was effectively implemented in the implementation process, which is beneficial to society. The results also indicate insufficient supervision over land-use violations while coping with conflicts between regional economic development change and general land-use planning. Therefore, the supervision and punishment of illegal land use should be strengthened by relevant departments to maintain the legal status of planning and to ensure the implementation of land-use control.

### 3.2. Adjustment of the General Land-Use Planning

#### 3.2.1. Analysis of Adjustment of the General Land-Use Plan

**Figure 4:**
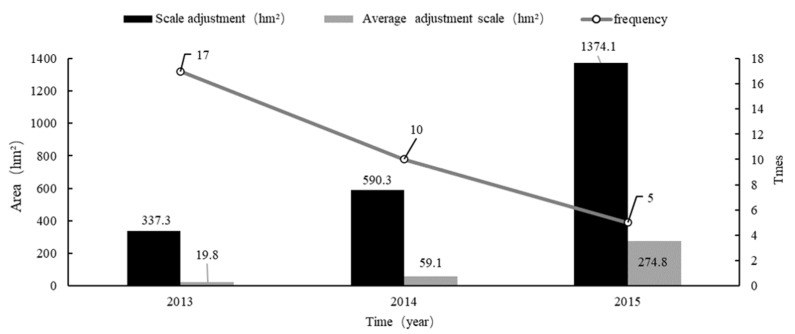
Planning adjustment in Rongchang District from 2013 to 2015.

**Figure 5:**
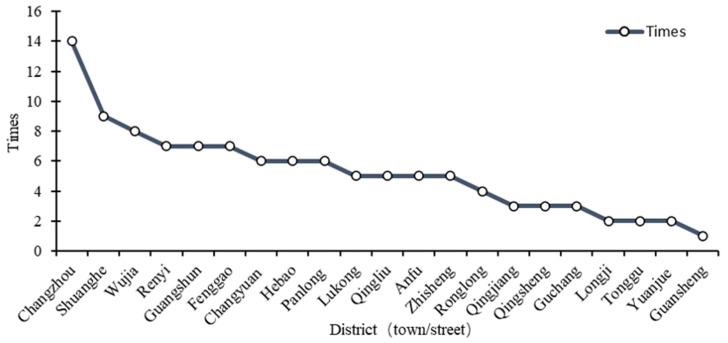
Adjustment frequency of general land-use planning in Rongchang District from 2013 to 2015.

A new round of general land-use planning for Rongchang District was completed in 2010. It was adjusted for the first time in 2013 and was adjusted 32 times from 2013 to 2015, with a total scale of 2301.7 hm^2^, a minimum scale of 0.03 hm^2^, and a maximum scale of 1245.75 hm^2^. The land-use types involved in the planning scheme adjustment cover a wide range of comprehensive adjustments to the construction land objective. The number and scale of adjustments in each year are shown in Figure 4. The distribution of planning adjustments in each town is shown in Figure 5. 

The data in Figure 4 show that the number and scale of adjustments each year are disordered. Specifically, the number of adjustments per year has decreased, while the average adjustment scale has increased. According to the analysis and summary in Table A1, most of the planning adjustments were in the form of project packaging, among which the single scale adjustment reached 1245.75 hm^2^ in 2015. This reflects the weakening of the legal status of planning.

The adjustment of planning in the study area from 2013 to 2015 involved 21 towns and streets in the area, with different adjustment scales among different regions. The total scale of the central urban area reached 1644.9 hm^2^, accounting for 70.9% of the total scale in the entire study area, mainly due to the frequent economic activities and rapid economic development of the streets in the central urban cities.

The data in Table 6 show a reduction of 113.4 hm^2^ of cultivated land index, which is mainly used to provide space for urban construction and expansion. The increase in urban land-use indicators is mainly due to the elastic surplus of planning, internal adjustment indicators, and additional indicators of construction land application approval. 

The analysis of the planning adjustment indicates that, in the case of the continuous development of the regional economy, it is necessary to adjust land-use planning to conduct planning management to address the uncertainties and emergencies of economic development. However, in reality, the general land-use plan is adjusted and modified frequently and in a disorderly way, which disrupts the general land-use plan in the process of land management.

#### 3.2.2. Consistency Analysis of Planning Adjustment

**Figure 6:**
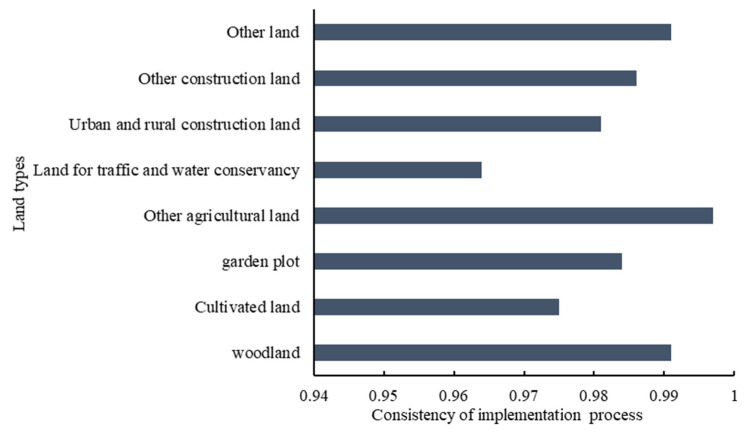
Spatial coincidence degrees and grades of implementation results of regional planning in Rongchang District in 2015.

The degree of spatial consistency of various types are shown in Figure 6.

Figure 6 shows that the adjustment of both arable land and urban and rural construction land in Rongchang District does not conform to planning. Except for the traffic and water utilization land, which is rated as grade E, the other land types are all within the interval of 0.95–1, which is rated as grade B. Therefore, to a large extent, the planning implementation results are in line with the planning, and the small gap indicates that there is still room for improvement in the future planning implementation period.

The degree of coincidence of cultivated land, garden land, forestland, and other land has been improved by comparing the spatial coincidence degree between the implementation process of the planning and the adjustment of the planning. Thus, the adjustment of the plan can alleviate the gap between implementation and planning caused by economic development and other uncertainties.

#### 3.2.3. Spatial Consistency of Planning Adjustment

There was no fixed time sequence or scale for the adjustment of land-use planning in the study area during the implementation process from 2013 to 2015 (Table A1). This reflects one-sidedness and a limitation to addressing the gap between planning and actual land demand due to the frequent and disorderly adjustment of planning, which underestimates its legal status.

**Figure 7:**
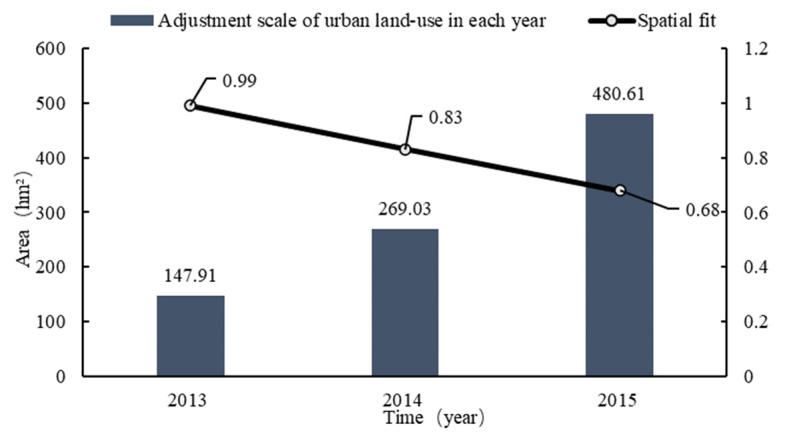
The spatial consistency between land-use change and land-use master plan adjustment.

The spatial coincidence degree between the planning adjustment data and land-use change data in Rongchang District in 2013 was as high as 0.99. The spatial coincidence degree decreased year by year, dropping to 0.83 in 2014 and as low as 0.68 in 2015, which indicates that the land-use change and the planning adjustment were highly consistent when the first adjustment was made two years after the implementation of the plan in 2013. On the one hand, planning should not be adjusted year after year. On the other hand, from the data shown in Figure 7, the degree of coincidence of the planning adjustment continually declines with the passage of time, and it is ideal for the degree to reach 0.99 after the implementation of planning two years later.

**Figure 8:**
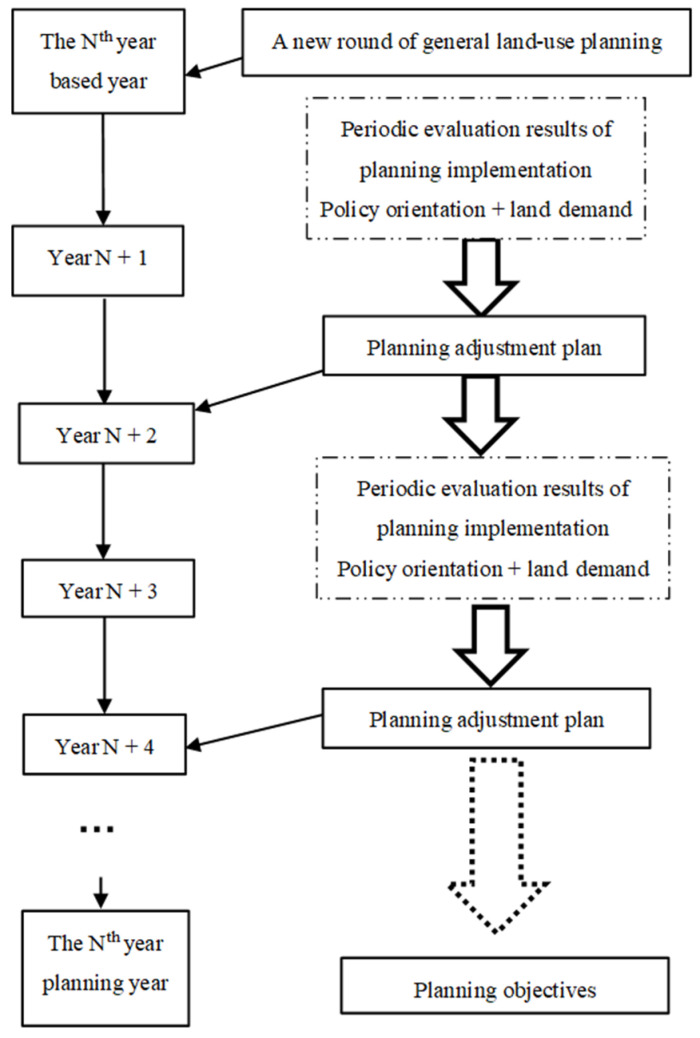
General plans for land-use periodic dynamic adjustment.

Based on the consistency of the research results, this paper chooses two years as a fixed time point to divide long-term planning into stages. The current policy orientation and land demand are combined to adjust planning in an orderly manner to construct a dynamic adjustment model with the aim of facilitating the guiding role of planning and ensuring its legal status without loss of the guiding role and economic benefits. The schematic diagram of periodic land-use dynamic adjustment is shown in Figure 8.

## 4. Discussion

(1) General land-use planning can effectively guide the land-use direction but cannot avoid the phenomenon of land demand exceeding the planning forecast.

The general land-use planning of Rongchang District has different controlling effects on different types of land use. It has the strongest protective effect on cultivated land, a certain restrictive effect on urban land expansion, and a weaker control effect on rural residential areas. China has always implemented the most stringent farmland protection system, and land management departments at all levels in China have strong motivation to firmly hold the red line of cultivated land and ensure national food security after experiencing the transformation idea of the protection policy of cultivated land from quantity to the trinity of “quantity, quality, and ecology” [53,54]. Therefore, the plan’s implementation of the arable land target must be given priority [55,56], and the economic development of a country is inseparable from the development of construction land. In recent years, the process of urbanization in China has been accelerating, a large amount of the agricultural population has moved to cities, and urban development has gradually accelerated. Thus, uncertainty brought about by the increasing demand for construction land is one of the main factors that affects planning control capabilities. The weak effect of planning on the exit control of rural settlements stems from the long-term lag of urbanization, in which the phenomenon of population flow exists without changes to the place of residence under the combined influence of China’s profound institutional and social characteristics.

Studies show that differences between planning and actual land needs are inevitable. On the one hand, planning is the study of an uncertain future, which belongs to the field of futurology [23]. It is impossible to fully predict economic and social development and an uncertain future [57]. On the other hand, general land-use planning in many districts in China does not focus on the supply of land resources and the internal demand of economic development; as a result, the content of the plan lacks scientific and forward-looking information. Therefore, deviations will always exist in the implementation progress.

(2) The guiding role of planning is largely attributed to continuous adjustments by departments at all levels during the implementation of planning.

General land-use planning is a long-term strategic planning that can be adjusted only by following the corresponding principles [58], and timely adjustments to planning can represent improvements. However, from 2013 to 2015, the planning adjustments of the study area were disordered and frequent, especially in places with rapid economic development. Superficially, this is the result of planning flexibility in the face of uncertain emergencies, which effectively alleviates the contradiction in land demand under economic development and changes in the short term. However, this occurs because the legal status of planning has not received adequate attention, and there is insufficient supervision of land-use behavior in relation to planning.

The development of the guidance function of planning comes at the expense of its authority. In this paper, two years was selected as the fixed time point for the dynamic adjustment of planning to reduce the number of planning adjustments and enhance the authority of planning. First, sufficient time is needed in the implementation process to solve the lag phenomenon between the change in land-use planning objectives and the change in land types. Over time, the scale of planning adjustments has increased year by year (Figure 4). If planning adjustments are made every year, it will increase the workload of various land management departments, and it is difficult to completely transform all large-scale land types. In modern society, where social and economic development is accelerating daily, the longer the implementation time of planning, the greater the deviation between the dynamic system of planning and actual land-use demand (Figure 7). Therefore, it is reasonable to choose two years as the time interval to adjust the plan. Considering other uncertain factors, this paper analyses the results of the implementation of the plan at the previous stage combined with the development requirements of the new era to provide ideas to safeguard both the economic benefits and the legal status of planning.

## 5. Conclusions

This study provides an efficient evaluation method for evaluating the implementation of general land-use planning by combining quantification and spatial location, which facilitates a more comprehensive evaluation of the implementation and guidance of planning. 

The empirical study showed that the planning has a certain guiding role and control ability for regional economic development, in which the effect of cultivated land, urban land, and rural residential land has gradually weakened and deviations consistently exits. By analyzing the adjustment situation of land-use planning in Rongchang District, this study finds that the adjustment is a response to regional economic development. The high degree of coincidence between planning adjustment and land use indicates that the guiding effect of planning is inseparable from planning adjustment, but its disorder and frequency challenge the authority of planning. We cannot accurately predict the long-term future situation, but short-term prediction is an effective way to guide the direction of land use in terms of time. Therefore, this paper considers two years as a time interval on the basis of the spatial coincidence degree to adjust land-use planning and realize the dialectical unity of the ‘dynamics’ and ‘determinacy’ of planning, aiming to maintain the rigidity and authority of planning while promoting sustainable economic and social development. Considering other uncertain factors, this paper chooses the evaluation results of the implementation of previous planning, policy guidance, and land demand for social and economic development in the next two years as references to construct a scientific and reasonable adjustment model (Figure 8). Increasing the ability of planning to cope with regional economic changes in an orderly way supports its legal status while facilitating its guiding role.

## Figures and Tables

**Table 1:** Change in land-use structure in Rongchang District, 2009–2015, and the planning control target in 2020.

First Class LT	LT	Year 2009/hm^2^	Proportion/%	Year 2015/hm^2^	Proportion/%	Area Changes from 2009 to 2015/hm^2^	Planning Control Target (Year 2020)	Proportion/%
Agricultural land	CL	58,631.84	54.45	59,381.77	55.15	749.93	58,281.69	64.06
	GP	3241.64	3.01	3194	2.97	−47.64	3054.62	3.36
	WL	15,118.03	14.04	15,022.84	13.95	−95.19	15,912.65	17.49
	OAL	13,225.67	12.28	11,896.5	11.05	−1329.17	13,725.81	15.09
	sum	90,217.18	83.78	89,495.11	83.12	−722.07	90,974.77	100
Construction land	UARCL	13,956.48	12.96	14,459.35	13.43	502.87	12,584.23	81.14
	LTWC	2100.96	1.95	2347.16	2.18	246.2	2820.64	18.19
	OICL	101.3	0.09	99.72	0.09	−1.58	103.54	0.67
	sum	16,158.74	15.01	16,906.23	15.7	747.49	15,508.41	100
Other land	W	1045.5	0.97	1030.52	0.96	−14.98	952.78	80.2
	NR	249.77	0.23	239.33	0.22	−10.44	235.23	19.8
	sum	1295.27	1.2	1269.85	1.18	−25.42	1188.01	100
summation		107,671.19	100	107,671.19	100	——	107,671.19	

Note: LT is land type; WL is woodland; CL is cultivated land; GP is garden plot; OAL is other agricultural land; UARCL is urban and rural construction land; LTWC is land for traffic and water conservancy; OICL is other independent construction land; OCL is other construction land; W is water; NR is nature reserve.

**Table 2:** Connotation of Speed indicators.

Name of Indicator	Definition of Indicator
Expansion strength/%	The expansion intensity is the ratio of the total change area of the land type to the total area of the land type in the assessment year. The greater the intensity of expansion, the greater the change in the land type during the implementation of the plan [44].
Use progress/%	The use progress of the indicator represents the ratio of the accumulated changed land area until the evaluation year to the total changed land area until the planned target year [44].

**Table 3:** Method and connotation of the spatial fit measurement model.

Table	Measurement Method	Connotation	Standard
Implementation process	$W_{i}=1-\frac{P_{\mathrm{ui}}}{P_{i}}\;\;\;\;\;\;\;\;\;\;$(2)	$W_{i}$ is the degree of spatial consistency of land i;${\;P}_{\mathrm{ui}}$ is the area of the plots that do not conform to the planning scheme among the plots with changes in land i during the planning implementation process from the base year to the end of the assessment;${\;P}_{i}$ represents the total planned area of land i [50]. Unit: hm^2^.	$W_{i}$ = 1 means in line with the plan; otherwise, not in line with it.
Implementation results	$W_{j}=\frac{N_{j}-P_{\mathrm{uj}}-{\mathrm{NP}}_{\mathrm{uj}}}{P_{j}}\;\;\;\;\;\;\;\;$(3)	$W_{j}$ is the spatial coincidence degree of land j in the planning implementation results; $N_{j}$ is the actual area for the assessment year; $P_{\mathrm{uj}}$ is the area that does not conform to the planning in the adjustment spot of land j in the evaluation year; ${\mathrm{NP}}_{\mathrm{uj}}$ is the unimplemented area of land j in the evaluation year; Pj is the planning area of land type j in the target year [50]. Unit: hm^2^.	For the consistency of the results, a grading standard with a difference of 0.05 is established. When $W_{i}$ = 1, it means level-A is completely consistent, and so on. It is divided into levels A–E.

**Table 4:** Residual situation of control objectives of general land-use planning in Rongchang District.

Land Types	Year 2009/hm^2^	Planning Control (Year 2020)	Year 2015/hm^2^	Planning Target Residual Rate/%
Cultivated land	58,631.84	58,281.69	59,381.77	−1.89
Urban industrial and mining land	2242.89	3583.69	3046.46	14.99
Rural residential land	11,711.83	8616.56	11,128.5	70.85

**Table 5:** Various types of land expansion and indicator utilization progress during the evaluation period in Rongchang District.

Index	Urban Industrial and Mining Land	Rural Residential Land	Cultivated Land
Expansion strength (%)	26.38	−5.24	1.26
Use progress (%)	59.93	18.85	None

**Table 6:** Adjustment and change in general land-use planning in Rongchang District from 2013 to 2015.

LT	WL	CL	GP	OAL	UL	RRL	ML	LTWC	OICL	OCL	W	NR
SA	−198.6	−113.4	−94.3	168.6	501.0	−364.5	25.5	102.5	−27.8	0.2	−8.8	9.7
I or D	D	D	D	I	I	D	I	I	D	D	D	D

Note: LT is land type; WL is woodland; CL is cultivated land; GP is garden plot; OAL is other agricultural land; UL is urban land; RRL is rural residential land; ML is mining land; LTWC is land for traffic and water conservancy; OICL is other independent construction land; OCL is other construction land; W is water; NR is nature reserve; D is decrease; I is increase; SA is scale adjustment.

## Data Availability

The data presented in this study are available on request from the first author.

## Appendix A

**Table A1:** Adjustment and Modification Project Statistics Table of General Land-Use Planning in Rongchang District from 2013 to 2015.

Project Number	Town/Streets Involved	Types of Adjustment	Land Type for Project	CL	GP	WL	OAL	UL	RRL	ML	OICL	LTWC	OCL	W	NR
20130308	Anfu	10	UR	−9.99	−1.57	0.00	−0.61	14.43	−2.26	0.00	0.00	0.00	0.00	0.00	0.00
20130308	Guangshun	10	UR	−35.14	−0.12	−0.52	−2.56	45.41	−6.96	0.00	0.00	0.00	−0.11	0.00	0.00
20130309	Changzhou	10	UR	−41.70	−1.35	−3.69	−0.85	56.22	−8.63	0.00	0.00	0.00	0.00	0.00	0.00
20130309	Fenggao	10	UR	−4.95	−0.47	−0.32	0.00	6.37	−0.63	0.00	0.00	0.00	0.00	0.00	0.00
20130310	Ronglong	10	UR	−4.00	0.00	−0.09	−0.09	4.50	−0.32	0.00	0.00	0.00	0.00	0.00	0.00
20130310	Ronglong	10	OICL	−12.09	0.00	−1.96	−0.14	0.00	−2.80	0.00	16.99	0.00	0.00	0.00	0.00
20130311	Anfu	10	HL	−7.20	0.00	0.00	−0.35	0.00	−0.56	0.00	0.00	8.11	0.00	0.00	0.00
20130312	Shuanghe	10	OICL	0.00	0.00	−0.04	0.00	0.00	0.00	0.00	0.04	0.00	0.00	0.00	0.00
20130313	Hebao	10	UR	−3.38	−0.97	0.00	0.00	5.70	−1.35	0.00	0.00	0.00	0.00	0.00	0.00
20130314	Panlong	10	UR	−0.31	0.00	0.00	0.00	0.37	−0.06	0.00	0.00	0.00	0.00	0.00	0.00
20130315	Changyuan	10	UR	−0.03	0.00	−0.44	0.00	0.47	0.00	0.00	0.00	0.00	0.00	0.00	0.00
20130316	Wujia	10	OICL	−0.14	0.00	0.00	0.00	0.00	−0.01	0.00	0.15	0.00	0.00	0.00	0.00
20130317	Shuanghe	10	OICL	−0.13	0.00	0.00	0.00	0.00	0.00	0.00	0.13	0.00	0.00	0.00	0.00
20130606	Guchang	10	UR	0.00	0.00	0.00	−0.24	0.24	0.00	0.00	0.00	0.00	0.00	0.00	0.00
20130606	Panlong	10	UR	−0.19	0.00	−0.35	0.00	0.54	0.00	0.00	0.00	0.00	0.00	0.00	0.00
20130606	Qingliu	10	UR	−0.21	0.00	0.00	0.00	0.21	0.00	0.00	0.00	0.00	0.00	0.00	0.00
20130606	Tonggu	10	UR	−0.33	0.00	0.00	0.00	0.35	−0.02	0.00	0.00	0.00	0.00	0.00	0.00
20130606	Yuanjue	10	UR	−0.28	0.00	0.00	0.00	0.30	−0.02	0.00	0.00	0.00	0.00	0.00	0.00
20130606	Zhisheng	10	UR	−0.18	0.00	0.00	0.00	0.18	0.00	0.00	0.00	0.00	0.00	0.00	0.00
20130606	Changzhou	10	OICL	−3.80	0.00	0.00	0.00	0.00	−0.42	0.00	4.22	0.00	0.00	0.00	0.00
20130606	Wujia	10	OICL	−0.65	0.00	0.00	0.00	0.00	0.00	0.00	0.65	0.00	0.00	0.00	0.00
20130608	Changzhou	10	HCL	0.00	0.00	−0.08	0.00	0.00	0.00	0.00	0.00	0.21	0.00	−0.13	0.00
20130608	Fenggao	10	HCL	−0.07	0.00	0.00	0.00	0.00	0.00	0.00	0.00	0.07	0.00	0.00	0.00
20130608	Qingjiang	10	HCL	−0.03	0.00	0.00	0.00	0.00	0.00	0.00	0.00	0.03	0.00	0.00	0.00
20130608	Qingliu	10	HCL	−0.05	0.00	0.00	0.00	0.00	0.00	0.00	0.00	0.05	0.00	0.00	0.00
20130608	Qingsheng	10	HCL	−0.09	0.00	0.00	0.00	0.00	0.00	0.00	0.00	0.09	0.00	0.00	0.00
20130608	Wujia	10	HCL	−0.03	0.00	0.00	0.00	0.00	0.00	0.00	0.00	0.03	0.00	0.00	0.00
20130609	Changyuan	10	UR	0.00	0.00	0.00	0.00	0.00	−0.30	0.00	0.30	0.00	0.00	0.00	0.00
20130609	Changzhou	10	OICL	0.00	0.00	−0.26	0.00	0.00	0.00	0.00	0.26	0.00	0.00	0.00	0.00
20130701	Hebao	10	ML	−0.71	0.00	0.00	−0.13	0.00	−0.01	0.85	0.00	0.00	0.00	0.00	0.00
20130714	Longji	10	UR	−0.11	0.00	0.00	0.00	0.11	0.00	0.00	0.00	0.00	0.00	0.00	0.00
20130714	Qingjiang	10	UR	−0.13	0.00	0.00	0.00	0.13	0.00	0.00	0.00	0.00	0.00	0.00	0.00
20130714	Qingliu	10	UR	−0.10	0.00	0.00	0.00	0.10	0.00	0.00	0.00	0.00	0.00	0.00	0.00
20130714	Renyi	10	UR	−0.05	0.00	0.00	−0.01	0.06	0.00	0.00	0.00	0.00	0.00	0.00	0.00
20130714	Ronglong	10	UR	−0.11	0.00	0.00	0.00	0.11	0.00	0.00	0.00	0.00	0.00	0.00	0.00
20130714	Wujia	10	OICL	−0.18	0.00	0.00	0.00	0.00	0.00	0.00	0.18	0.00	0.00	0.00	0.00
20130901	Changzhou	10	UR	−0.76	0.00	−0.86	0.00	1.81	−0.19	0.00	0.00	0.00	0.00	0.00	0.00
20130901	Lukong	10	OICL	0.00	0.00	−1.02	0.00	0.00	0.00	0.00	1.02	0.00	0.00	0.00	0.00
20131028	Changzhou	10	UR	−8.55	0.00	−1.02	0.00	10.21	−0.64	0.00	0.00	0.00	0.00	0.00	0.00
20131028	Lukong	10	UR	−0.09	0.00	0.00	0.00	0.09	0.00	0.00	0.00	0.00	0.00	0.00	0.00
20131028	Changzhou	10	HL	−15.29	0.00	−4.26	−0.72	0.00	−2.38	0.00	0.00	22.65	0.00	0.00	0.00
20131028	Lukong	10	HL	−0.94	0.00	−0.13	0.00	0.00	−0.05	0.00	0.00	1.12	0.00	0.00	0.00
20131028	Changzhou	10	OICL	0.00	0.00	0.00	0.00	0.00	−0.10	0.00	0.10	0.00	0.00	0.00	0.00
20140108	Guangshun	10	UR	−40.33	−0.03	−0.75	−0.58	50.27	−8.44	−0.14	0.00	0.00	0.00	0.00	0.00
20140114	Shuanghe	10	HL	0.00	0.00	0.00	0.00	0.00	−0.03	0.00	0.00	0.03	0.00	0.00	0.00
20140501	Changzhou	10	HCL	−2.97	0.00	−5.54	−0.41	0.00	−0.66	0.00	0.00	9.59	0.00	−0.01	0.00
20140501	Lukong	10	HCL	−2.12	−0.13	−5.02	−0.47	0.00	−0.25	0.00	0.00	8.19	0.00	−0.20	0.00
20140710	Changzhou	10	OICL	−0.56	0.00	0.00	−0.17	0.00	0.00	0.00	0.73	0.00	0.00	0.00	0.00
20140830	Anfu	10	UR	−14.59	−1.81	−0.86	−3.51	23.56	−2.79	0.00	0.00	0.00	0.00	0.00	0.00
20140830	Changzhou	10	UR	0.00	0.00	0.00	0.00	8.33	0.00	0.00	0.00	0.00	0.00	−8.33	0.00
20140830	Fenggao	10	UR	0.00	0.00	0.00	0.00	0.18	0.00	0.00	0.00	0.00	0.00	−0.18	0.00
20140830	Guangshun	10	UR	−43.91	0.00	−3.36	−23.50	79.44	−8.58	−0.09	0.00	0.00	0.00	0.00	0.00
20140830	Hebao	10	UR	−0.52	0.00	0.00	0.00	0.52	0.00	0.00	0.00	0.00	0.00	0.00	0.00
20140830	Lukong	10	UR	−3.30	0.00	−0.18	−0.57	4.56	−0.51	0.00	0.00	0.00	0.00	0.00	0.00
20140830	Panlong	10	UR	−5.12	0.00	0.00	−0.82	8.24	−1.21	0.00	0.00	−1.09	0.00	0.00	0.00
20140830	Renyi	10	UR	−1.20	0.00	−0.12	−0.22	1.54	0.00	0.00	0.00	0.00	0.00	0.00	0.00
20140830	Ronglong	10	UR	−52.11	−0.83	−12.00	−10.09	81.71	−6.68	0.00	0.00	0.00	0.00	0.00	0.00
20140830	Wujia	10	UR	−0.34	0.00	0.00	−1.17	9.69	−8.18	0.00	0.00	0.00	0.00	0.00	0.00
20140830	Ronglong	10	WL	−12.31	−0.08	14.78	−2.16	0.00	−0.23	0.00	0.00	0.00	0.00	0.00	0.00
20140920	Panlong	10	RRL	−1.55	0.00	−0.08	−0.31	0.00	2.01	0.00	0.00	0.00	−0.07	0.00	0.00
20140920	Shuanghe	10	RRL	−0.35	0.00	0.00	−0.96	0.00	1.31	0.00	0.00	0.00	0.00	0.00	0.00
20141010	Changyuan	10	UR	−0.03	0.00	−0.03	0.00	0.06	0.00	0.00	0.00	0.00	0.00	0.00	0.00
20141018	Anfu	10	HL	−6.38	0.00	−0.41	−1.58	0.00	−1.38	0.00	0.00	9.75	0.00	0.00	0.00
20141018	Renyi	10	HL	−1.83	−0.59	0.00	−0.44	0.00	−0.35	0.00	0.00	3.21	0.00	0.00	0.00
20141018	Wujia	10	HL	−1.22	0.00	0.00	−0.41	0.00	0.00	0.00	0.00	1.63	0.00	0.00	0.00
20141019	Qingliu	10	UR	−0.58	0.00	0.00	−0.12	0.74	−0.04	0.00	0.00	0.00	0.00	0.00	0.00
20141123	Zhisheng	10	UR	−0.17	0.00	0.00	−0.02	0.19	0.00	0.00	0.00	0.00	0.00	0.00	0.00
20150203	Wujia	10	ML	−1.06	0.00	0.00	−0.43	0.00	0.00	1.49	0.00	0.00	0.00	0.00	0.00
20150203	Anfu	10	UR	−12.96	0.00	−0.18	−1.88	16.93	−1.72	0.00	0.00	−0.19	0.00	0.00	0.00
20150203	Changyuan	10	UR	0.00	0.00	−1.53	−0.05	1.58	0.00	0.00	0.00	0.00	0.00	0.00	0.00
20150203	Changzhou	10	UR	−64.26	−13.30	−22.10	−16.67	137.10	−8.59	−2.68	0.00	−8.92	−0.16	−0.42	0.00
20150203	Fenggao	10	UR	−1.31	0.00	−23.64	−0.38	25.40	0.00	−0.07	0.00	0.00	0.00	0.00	0.00
20150203	Guchang	10	UR	−0.49	0.00	−0.04	−0.20	0.73	0.00	0.00	0.00	0.00	0.00	0.00	0.00
20150203	Guangshun	10	UR	−45.60	−0.63	−3.56	−10.42	66.53	−6.32	0.00	0.00	0.00	0.00	0.00	0.00
20150203	Hebao	10	UR	−1.73	−0.01	−0.20	−0.20	2.86	−0.72	0.00	0.00	0.00	0.00	0.00	0.00
20150203	Longji	10	UR	−0.54	0.00	0.00	−0.09	0.64	−0.01	0.00	0.00	0.00	0.00	0.00	0.00
20150203	Lukong	10	UR	−18.33	−0.33	−15.39	−5.60	44.76	−2.20	0.00	−2.91	0.00	0.00	0.00	0.00
20150203	Panlong	10	UR	−2.72	0.00	0.00	−0.80	4.19	−0.39	0.00	0.00	−0.28	0.00	0.00	0.00
20150203	Qingsheng	10	UR	−2.53	0.00	0.00	−0.56	3.09	0.00	0.00	0.00	0.00	0.00	0.00	0.00
20150203	Renyi	10	UR	−3.34	−1.75	−0.07	−1.38	6.96	−0.32	0.00	0.00	−0.10	0.00	0.00	0.00
20150203	Ronglong	10	UR	−9.83	−0.28	−2.72	−2.08	48.41	−1.51	0.00	−31.99	0.00	0.00	0.00	0.00
20150203	Wujia	10	UR	−2.67	0.00	0.00	−0.30	3.14	−0.17	0.00	0.00	0.00	0.00	0.00	0.00
20150203	Zhisheng	10	UR	−4.51	−1.74	−0.10	−0.90	7.82	−0.57	0.00	0.00	0.00	0.00	0.00	0.00
20150203	Anfu	10	HL	−5.90	0.00	0.00	−1.83	0.00	−1.72	0.00	0.00	9.45	0.00	0.00	0.00
20150203	Changzhou	10	HL	−14.17	−0.48	−5.81	−4.45	0.00	−4.48	−0.90	0.00	30.29	0.00	0.00	0.00
20150203	Guangshun	10	HL	−0.59	0.00	0.00	−0.24	0.00	−2.17	0.00	0.00	3.00	0.00	0.00	0.00
20150203	Panlong	10	HL	−0.75	0.00	−0.01	−0.12	0.00	0.00	0.00	0.00	0.88	0.00	0.00	0.00
20150203	Anfu	10	RRL	−0.47	0.00	0.00	−0.11	0.00	0.58	0.00	0.00	0.00	0.00	0.00	0.00
20150203	Changyuan	10	RRL	−4.08	−1.58	−0.74	−0.90	0.00	7.30	0.00	0.00	0.00	0.00	0.00	0.00
20150203	Fenggao	10	RRL	−3.92	−0.92	−0.50	−1.48	0.00	6.89	0.00	0.00	−0.07	0.00	0.00	0.00
20150203	Guchang	10	RRL	−0.99	−0.06	−0.31	−0.67	0.00	2.03	0.00	0.00	0.00	0.00	0.00	0.00
20150203	Guangshun	10	RRL	−5.61	0.00	−1.52	−1.14	0.00	8.27	0.00	0.00	0.00	0.00	0.00	0.00
20150203	Hebao	10	RRL	−5.00	0.00	−0.12	−1.29	0.00	6.41	0.00	0.00	0.00	0.00	0.00	0.00
20150203	Longji	10	RRL	−4.31	−0.16	−0.18	−0.93	0.00	5.58	0.00	0.00	0.00	0.00	0.00	0.00
20150203	Lukong	10	RRL	−1.05	−0.27	−1.42	−0.27	0.00	3.01	0.00	0.00	0.00	0.00	0.00	0.00
20150203	Qingjiang	10	RRL	−1.01	0.00	0.00	−0.19	0.00	1.20	0.00	0.00	0.00	0.00	0.00	0.00
20150203	Renyi	10	RRL	−0.25	0.00	0.00	−0.10	0.00	0.35	0.00	0.00	0.00	0.00	0.00	0.00
20150203	Ronglong	10	RRL	−3.55	−0.37	−1.73	−2.68	0.00	8.80	0.00	−0.47	0.00	0.00	0.00	0.00
20150203	Shuanghe	10	RRL	−0.63	0.00	−0.41	−0.16	0.00	1.20	0.00	0.00	0.00	0.00	0.00	0.00
20150203	Tonggu	10	RRL	−0.32	0.00	0.00	−0.10	0.00	0.42	0.00	0.00	0.00	0.00	0.00	0.00
20150203	Yuanjue	10	RRL	−0.16	0.00	−0.78	−3.18	0.00	4.12	0.00	0.00	0.00	0.00	0.00	0.00
20150203	Guansheng	10	OICL	−0.08	0.00	0.00	−0.02	0.00	−0.07	0.00	0.17	0.00	0.00	0.00	0.00
20150203	Longji	10	OICL	−0.10	0.00	0.00	−0.02	0.00	0.00	0.00	0.12	0.00	0.00	0.00	0.00
20150203	Lukong	10	OICL	0.00	−0.01	−1.07	−0.04	0.00	0.00	0.00	1.30	−0.18	0.00	0.00	0.00
20150203	Qingliu	10	OICL	0.00	−0.07	0.00	0.00	0.00	−0.07	0.00	0.14	0.00	0.00	0.00	0.00
20150203	Qingsheng	10	UR	−1.96	−3.11	−6.21	−0.50	0.00	−0.22	0.00	12.00	0.00	0.00	0.00	0.00
20150203	Renyi	10	OICL	−0.19	0.00	0.00	−0.06	0.00	0.00	0.00	0.25	0.00	0.00	0.00	0.00
20150203	Ronglong	10	OICL	−1.39	0.00	−1.58	−0.35	0.00	0.00	0.00	3.32	0.00	0.00	0.00	0.00
20150203	Shuanghe	10	OICL	0.00	0.00	−0.11	−0.01	0.00	−0.85	0.00	0.97	0.00	0.00	0.00	0.00
20150203	Wujia	10	OICL	0.00	0.00	0.00	0.00	0.00	−0.07	0.00	0.07	0.00	0.00	0.00	0.00
20150203	Fenggao	10	FAL	0.00	0.00	−12.25	12.25	0.00	0.00	0.00	0.00	0.00	0.00	0.00	0.00
20150203	Guansheng	10	FAL	−15.54	−0.11	−4.89	20.54	0.00	0.00	0.00	0.00	0.00	0.00	0.00	0.00
20150203	Hebao	10	FAL	−14.30	0.00	−6.36	20.66	0.00	0.00	0.00	0.00	0.00	0.00	0.00	0.00
20150203	Panlong	10	FAL	−19.01	0.00	−4.67	23.68	0.00	0.00	0.00	0.00	0.00	0.00	0.00	0.00
20150203	Qingliu	10	FAL	−0.60	−4.30	−16.59	21.49	0.00	0.00	0.00	0.00	0.00	0.00	0.00	0.00
20150203	Renyi	10	FAL	−4.40	0.00	−4.21	8.61	0.00	0.00	0.00	0.00	0.00	0.00	0.00	0.00
20150203	Ronglong	10	FAL	−4.82	−22.74	−25.54	53.52	0.00	−0.42	0.00	0.00	0.00	0.00	0.00	0.00
20150203	Shuanghe	10	FAL	−1.99	−9.81	−17.79	30.18	0.00	−0.30	−0.29	0.00	0.00	0.00	0.00	0.00
20150203	Tonggu	10	FAL	−6.53	−5.56	−1.43	13.52	0.00	0.00	0.00	0.00	0.00	0.00	0.00	0.00
20150203	Wujia	10	FAL	−2.54	−8.46	−2.20	13.20	0.00	0.00	0.00	0.00	0.00	0.00	0.00	0.00
20150203	Yuanjue	10	FAL	−4.77	0.00	−9.50	14.27	0.00	0.00	0.00	0.00	0.00	0.00	0.00	0.00
20150203	Changzhou	10	HCL	−3.80	−0.73	−4.28	−0.85	0.00	−0.06	0.00	0.00	11.33	0.00	−1.61	0.00
20150203	Lukong	10	HCL	−6.90	0.00	−1.81	−1.51	0.00	−0.94	0.00	0.00	11.16	0.00	0.00	0.00
20150203	Changzhou	10	RS	−1.00	−0.01	−0.49	−0.28	−1.55	0.00	0.00	0.00	−0.05	0.00	3.38	0.00
20150203	Zhisheng	10	RS	−1.24	−0.18	−1.05	−0.47	0.00	0.00	−0.11	0.00	0.00	0.00	3.39	−0.34
20150510	Hebao	10	OICL	−0.20	0.00	0.00	−0.05	0.00	0.00	0.00	0.25	0.00	0.00	0.00	0.00
20150512	Guangshun	10	OICL	−0.02	0.00	0.00	−0.01	0.00	0.00	0.00	0.03	0.00	0.00	0.00	0.00
20150512	Renyi	10	OICL	−0.12	0.00	−0.01	−0.03	0.00	0.00	0.00	0.16	0.00	0.00	0.00	0.00
20150701	Renyi	10	ML	−0.15	0.00	0.00	−0.03	0.00	0.00	0.18	0.00	0.00	0.00	0.00	0.00
20150811	Changzhou	10	UR	−39.17	−21.25	−9.24	−14.09	90.39	−6.43	0.00	0.00	0.00	−0.21	0.00	0.00
20150811	Panlong	10	UR	−12.05	−0.27	−0.09	−2.25	15.17	−0.51	0.00	0.00	0.00	0.00	0.00	0.00
20150811	Zhisheng	10	UR	−0.43	−1.24	−2.29	−0.95	4.91	0.00	0.00	0.00	0.00	0.00	0.00	0.00
20130308	Changzhou	20	UR	18.44	1.90	2.28	3.44	−27.92	1.86	0.00	0.00	0.00	0.00	0.00	0.00
20130308	Guangshun	20	UR	16.88	0.17	0.00	3.03	−31.67	11.13	0.00	0.00	0.30	0.16	0.00	0.00
20130309	Guangshun	20	UR	17.56	0.00	0.15	2.96	−23.71	2.95	0.00	0.00	0.00	0.09	0.00	0.00
20130310	Fenggao	20	UR	7.35	0.00	2.49	1.37	−13.25	2.04	0.00	0.00	0.00	0.00	0.00	0.00
20130310	Ronglong	20	OICL	2.31	0.00	1.52	0.89	0.00	2.86	0.00	−7.58	0.00	0.00	0.00	0.00
20130312	Shuanghe	20	UR	0.00	0.00	0.01	0.00	−0.01	0.00	0.00	0.00	0.00	0.00	0.00	0.00
20130312	Shuanghe	20	OICL	0.00	0.00	0.03	0.00	0.00	0.00	0.00	−0.03	0.00	0.00	0.00	0.00
20130313	Hebao	20	UR	0.41	0.00	0.00	0.01	−5.73	5.31	0.00	0.00	0.00	0.00	0.00	0.00
20130314	Panlong	20	UR	0.32	0.00	0.00	0.05	−0.37	0.00	0.00	0.00	0.00	0.00	0.00	0.00
20130315	Shuanghe	20	UR	0.35	0.00	0.05	0.07	−0.47	0.00	0.00	0.00	0.00	0.00	0.00	0.00
20130316	Shuanghe	20	UR	0.00	0.00	0.15	0.00	−0.15	0.00	0.00	0.00	0.00	0.00	0.00	0.00
20130317	Shuanghe	20	UR	0.04	0.00	0.08	0.01	−0.13	0.00	0.00	0.00	0.00	0.00	0.00	0.00
20130606	Fenggao	20	UR	3.03	1.14	1.31	0.56	−6.66	0.62	0.00	0.00	0.00	0.00	0.00	0.00
20130609	Changzhou	20	UR	0.22	0.00	0.30	0.04	−0.56	0.00	0.00	0.00	0.00	0.00	0.00	0.00
20130714	Fenggao	20	UR	0.58	0.00	0.00	0.11	−0.69	0.00	0.00	0.00	0.00	0.00	0.00	0.00
20130901	Changzhou	20	UR	0.73	0.81	0.00	0.14	−1.83	0.15	0.00	0.00	0.00	0.00	0.00	0.00
20130901	Lukong	20	UR	0.81	0.00	0.00	0.14	−1.01	0.06	0.00	0.00	0.00	0.00	0.00	0.00
20131028	Shuanghe	20	UR	2.83	0.00	0.35	0.44	−4.31	0.59	0.00	0.00	0.00	0.10	0.00	0.00
20131028	Changzhou	20	OICL	3.44	0.23	0.41	0.81	0.00	0.43	0.00	−6.10	0.00	0.09	0.00	0.69
20140108	Changyuan	20	UR	21.48	0.40	5.60	3.71	−38.14	6.95	0.00	0.00	0.00	0.00	0.00	0.00
20140108	Guangshun	20	UR	7.12	0.31	0.69	1.31	−12.15	2.72	0.00	0.00	0.00	0.00	0.00	0.00
20140710	Changzhou	20	OICL	0.63	0.00	0.00	0.09	0.00	0.00	0.00	−0.72	0.00	0.00	0.00	0.00
20140830	Anfu	20	UR	14.32	0.00	1.03	3.44	−28.62	9.83	0.00	0.00	0.00	0.00	0.00	0.00
20140830	Changyuan	20	UR	0.52	0.00	1.28	0.10	−2.17	0.27	0.00	0.00	0.00	0.00	0.00	0.00
20140830	Changzhou	20	UR	42.97	4.12	5.83	11.62	−75.21	10.58	0.00	0.00	0.00	0.09	0.00	0.00
20140830	Fenggao	20	UR	8.21	0.09	0.65	2.17	−12.60	1.30	0.00	0.00	0.00	0.18	0.00	0.00
20140830	Guchang	20	UR	3.12	0.00	0.08	0.58	−4.94	1.16	0.00	0.00	0.00	0.00	0.00	0.00
20140830	Guangshun	20	UR	0.00	0.00	0.00	0.14	−20.67	0.00	20.53	0.00	0.00	0.00	0.00	0.00
20140830	Hebao	20	UR	1.22	0.01	0.13	0.29	−3.66	2.01	0.00	0.00	0.00	0.00	0.00	0.00
20140830	Lukong	20	UR	4.45	0.00	0.46	0.87	−6.23	0.45	0.00	0.00	0.00	0.00	0.00	0.00
20140830	Panlong	20	UR	5.53	0.17	0.00	1.37	−8.22	1.15	0.00	0.00	0.00	0.00	0.00	0.00
20140830	Qingsheng	20	UR	2.86	0.00	0.00	0.97	−4.65	0.35	0.47	0.00	0.00	0.00	0.00	0.00
20140830	Renyi	20	UR	0.96	0.81	0.00	0.18	−2.18	0.23	0.00	0.00	0.00	0.00	0.00	0.00
20140830	Shuanghe	20	UR	14.50	0.00	2.29	3.99	−26.30	5.52	0.00	0.00	0.00	0.00	0.00	0.00
20140830	Zhisheng	20	UR	1.20	0.00	0.05	0.29	−1.54	0.00	0.00	0.00	0.00	0.00	0.00	0.00
20140830	Guangshun	20	OICL	3.87	0.26	4.65	1.52	0.00	2.44	4.92	−17.66	0.00	0.00	0.00	0.00
20140830	Lukong	20	OICL	1.64	0.00	0.42	0.51	0.00	0.61	0.00	−3.18	0.00	0.00	0.00	0.00
20141010	Changyuan	20	UR	0.21	0.00	0.00	0.04	−0.25	0.00	0.00	0.00	0.00	0.00	0.00	0.00
20141019	Changzhou	20	UR	0.57	0.00	0.05	0.13	−0.75	0.00	0.00	0.00	0.00	0.00	0.00	0.00
20141123	Zhisheng	20	UR	0.00	0.00	0.00	0.00	−0.19	0.19	0.00	0.00	0.00	0.00	0.00	0.00
20150203	Changzhou	20	UR	0.72	0.00	1.28	0.12	−3.32	1.20	0.00	0.00	0.00	0.00	0.00	0.00
20150203	Fenggao	20	UR	0.00	0.00	1.08	1.13	−2.41	0.20	0.00	0.00	0.00	0.00	0.00	0.00
20150203	Panlong	20	UR	1.53	0.00	0.00	0.23	−1.76	0.00	0.00	0.00	0.00	0.00	0.00	0.00
20150203	Anfu	20	HL	2.12	0.00	0.23	0.46	0.00	1.01	0.00	0.00	−3.82	0.00	0.00	0.00
20150203	Changzhou	20	HL	4.14	0.30	1.16	2.00	0.00	1.15	0.61	0.00	−9.36	0.00	0.00	0.00
20150203	Lukong	20	HL	0.01	0.00	0.00	0.00	0.00	0.00	0.00	0.00	−0.01	0.00	0.00	0.00
20150203	Panlong	20	HL	2.22	0.00	0.00	0.33	0.00	0.00	0.00	0.00	−2.55	0.00	0.00	0.00
20150203	Anfu	20	RRL	22.50	0.00	0.00	4.26	0.00	−26.76	0.00	0.00	0.00	0.00	0.00	0.00
20150203	Changyuan	20	RRL	16.62	0.00	0.00	3.24	0.00	−19.86	0.00	0.00	0.00	0.00	0.00	0.00
20150203	Changzhou	20	RRL	11.53	0.00	0.28	2.13	0.00	−13.94	0.00	0.00	0.00	0.00	0.00	0.00
20150203	Fenggao	20	RRL	16.52	0.00	0.68	3.03	0.00	−20.23	0.00	0.00	0.00	0.00	0.00	0.00
20150203	Guchang	20	RRL	16.88	0.00	0.00	3.17	0.00	−20.05	0.00	0.00	0.00	0.00	0.00	0.00
20150203	Guansheng	20	RRL	6.36	0.00	0.00	1.29	0.00	−7.65	0.00	0.00	0.00	0.00	0.00	0.00
20150203	Guangshun	20	RRL	8.18	0.00	0.00	1.73	0.00	−9.91	0.00	0.00	0.00	0.00	0.00	0.00
20150203	Hebao	20	RRL	23.68	0.00	0.00	4.43	0.00	−28.11	0.00	0.00	0.00	0.00	0.00	0.00
20150203	Longji	20	RRL	8.86	0.00	0.00	1.64	0.00	−10.50	0.00	0.00	0.00	0.00	0.00	0.00
20150203	Lukong	20	RRL	5.97	0.00	0.00	1.11	0.00	−7.08	0.00	0.00	0.00	0.00	0.00	0.00
20150203	Panlong	20	RRL	23.50	0.00	0.00	4.39	0.00	−27.89	0.00	0.00	0.00	0.00	0.00	0.00
20150203	Qingjiang	20	RRL	12.87	0.00	0.00	2.39	0.00	−15.26	0.00	0.00	0.00	0.00	0.00	0.00
20150203	Qingliu	20	RRL	15.66	0.00	0.00	2.81	0.00	−18.47	0.00	0.00	0.00	0.00	0.00	0.00
20150203	Qingsheng	20	RRL	11.86	0.00	0.00	2.23	0.00	−14.09	0.00	0.00	0.00	0.00	0.00	0.00
20150203	Renyi	20	RRL	11.94	0.00	0.00	2.19	0.00	−14.13	0.00	0.00	0.00	0.00	0.00	0.00
20150203	Ronglong	20	RRL	54.17	0.00	0.92	9.98	0.00	−65.13	0.06	0.00	0.00	0.00	0.00	0.00
20150203	Shuanghe	20	RRL	22.82	0.00	0.00	4.35	0.00	−27.17	0.00	0.00	0.00	0.00	0.00	0.00
20150203	Tonggu	20	RRL	4.70	0.00	0.00	0.85	0.00	−5.55	0.00	0.00	0.00	0.00	0.00	0.00
20150203	Wujia	20	RRL	14.09	0.00	0.00	2.69	0.00	−16.78	0.00	0.00	0.00	0.00	0.00	0.00
20150203	Yuanjue	20	RRL	12.96	0.00	2.49	2.42	0.00	−17.87	0.00	0.00	0.00	0.00	0.00	0.00
20150203	Zhisheng	20	RRL	9.06	0.00	0.00	1.74	0.00	−10.80	0.00	0.00	0.00	0.00	0.00	0.00
20150203	Qingsheng	20	OICL	0.45	0.00	0.00	0.08	0.00	0.14	0.00	−0.67	0.00	0.00	0.00	0.00
20150203	Fenggao	20	FAL	2.34	0.00	0.00	−2.34	0.00	0.00	0.00	0.00	0.00	0.00	0.00	0.00
20150203	Panlong	20	FAL	23.49	0.00	3.17	−36.64	0.00	0.65	0.00	0.00	0.00	0.00	0.00	9.33
20150203	Yuanjue	20	FAL	3.80	0.00	0.00	−3.80	0.00	0.00	0.00	0.00	0.00	0.00	0.00	0.00
20150203	Changzhou	20	HCL	0.00	0.00	0.00	0.00	0.00	0.00	0.00	0.00	0.00	0.00	0.00	0.00
20150203	Changzhou	20	RS	1.43	0.00	0.97	0.32	0.00	0.00	0.00	0.00	0.00	0.00	−2.72	0.00
20150203	Zhisheng	20	RS	0.90	0.15	0.55	0.12	0.00	0.18	0.08	0.00	0.00	0.00	−1.98	0.00
20150811	Changzhou	20	UR	1.59	0.50	0.87	0.33	−3.31	0.02	0.00	0.00	0.00	0.00	0.00	0.00
20150811	Guangshun	20	UR	7.02	0.02	0.33	1.34	−9.95	1.24	0.00	0.00	0.00	0.00	0.00	0.00
20150811	Renyi	20	UR	0.96	0.71	0.00	0.17	−1.92	0.08	0.00	0.00	0.00	0.00	0.00	0.00
20150811	Zhisheng	20	UR	0.86	0.99	0.03	0.20	−2.11	0.03	0.00	0.00	0.00	0.00	0.00	0.00

Note: WL is woodland; CL is cultivated land; GP is garden plot; OAL is other agricultural land; UL is urban land; RRL is rural residential land; ML is mining land; LTWC is land for traffic and water conservancy; OICL is other independent construction land; HCL is hydraulic construction land; HL is highway land; ML is mining land; FAL is facility agricultural land; RS is reservoir surface; OCL is other construction land; W is water; NR is nature reserve.
